# Systems Pharmacogenomic Landscape of Drug Similarities from LINCS data: Drug Association Networks

**DOI:** 10.1038/s41598-019-44291-3

**Published:** 2019-05-24

**Authors:** Aliyu Musa, Shailesh Tripathi, Matthias Dehmer, Olli Yli-Harja, Stuart A. Kauffman, Frank Emmert-Streib

**Affiliations:** 1 Predictive Society and Data Analytics Lab, Tampere University, Tampere, Korkeakoulunkatu 10, 33720 Tampere, Finland; 2 Institute of Biosciences and Medical Technology, Tampere University, Tampere, Korkeakoulunkatu 10, 33720 Tampere, Finland; 3 Department for Biomedical Computer Science and Mechatronics, UMIT - The Health and Lifesciences University, Eduard Wallnoefer Zentrum 1, 6060 Hall in Tyrol, Austria; 4 College of Computer and Control Engineering, Nankai University, Tianjin, 300350 P.R. China; 5 Computational Systems Biology Lab, Tampere University of Technology, Korkeakoulunkatu 10, 33720 Tampere, Finland; 6 Institute for Intelligent Production, Faculty for Management, University of Applied Sciences Upper Austria, Wehrgrabengasse 1-3, 4400 Steyr, Austria; 7 Institute for Systems Biology, Seattle, WA 98109 USA

**Keywords:** Computational biology and bioinformatics, Mathematics and computing

## Abstract

Modern research in the biomedical sciences is data-driven utilizing high-throughput technologies to generate big genomic data. The Library of Integrated Network-based Cellular Signatures (LINCS) is an example for a large-scale genomic data repository providing hundred thousands of high-dimensional gene expression measurements for thousands of drugs and dozens of cell lines. However, the remaining challenge is how to use these data effectively for pharmacogenomics. In this paper, we use LINCS data to construct drug association networks (DANs) representing the relationships between drugs. By using the Anatomical Therapeutic Chemical (ATC) classification of drugs we demonstrate that the DANs represent a systems pharmacogenomic landscape of drugs summarizing the entire LINCS repository on a genomic scale meaningfully. Here we identify the modules of the DANs as therapeutic attractors of the ATC drug classes.

## Introduction

Recent availability of large-scale pharmacogenomic data have presented new opportunities but also challanges for tailored patient treatment, drug design and drug safety^1,2^. Vast efforts have been placed into discovering the drug mode-of-action (MoA) and understanding the genetic interactions within cells for disease treatment^3^. Importantly, it has been found that drug-induced transcriptional profiles from cell lines can be used to characterize therapeutic effects, enabling new computational ways for pharmacogenomics for identifying small drug molecules, compounds and drug-drug similarities solely based on gene expression profiles^4–7^.

The Library of Integrated Network-based Cellular Signatures (LINCS) program^8^, (https://clue.io/), funded by the Big Data to Knowledge (BD2K) Initiative at the National Institutes of Health (NIH), generated genetic and molecular signatures of human cell lines in response to various perturbations. The LINCS data repository is a vast library of gene expression profiles covering seventy-two human cell lines and include experiments for thousands of chemical perturbagens (small drug molecules), and drugs added to the cell cultures to induce changes in the gene expression profiles. The LINCS data are publicly available from the Gene Expression Omnibus (GEO) database. Based on these data, several advanced computational methods have been proposed for drug repurposing, identification of mode-of-action (MoA) and discovering phenotypic relations^9–11^; for an overview see^12^. The reason why gene expression data can be utilized as surrogates for the structure of chemical compounds to study mechanism of action and phenotypic impact between compounds^13–17^ it that in^18^ it has been shown that structurally similar compounds have similar gene expression profiles, furthermore compounds with similar gene expression signatures tend to interact with similar protein targets^19^.

Traditionally, pharmacology approaches focus on single drugs at a time to study their action, effects or safety^20^. This is similar to traditional molecular biology approaches that focused on single genes or proteins^21^. However, due to modern genomic high-throuhgput technologies, nowadays, it is possible to study many genes or proteins simultenously^22^. Pharmacogenomics and Systems Pharmacogenomics aim to utilize such genomic profiles to expand beyond single drugs^23^. For instance, in^24^ drug-target and drug-drug networks have been constructed based on the DrugBank database utilizing information about FDA approved and non-approved drugs and their corresponding targets. However, their analysis focused exclusively on drugs and compounds with known targets and did not take into consideration dynamic activity profiles as represented, e.g., by transcriptomics data. In^25^ some disadvantages were avoided by using gene expression profiles for which Pearson correlation-based networks were constructed. A problem is that the used data were generated from many independent, uncoordinated laboratories using varying platforms and samle preprations. Another drawback of this study is the small number of used profiles (<7,000) and the very limited number of studied drugs (~200). Similar data were used in^4,17^ but the construction of the drug network differed. Also, their analysis focused on drugs with known MoA. A different approach has been taken in^26^ where a drug-drug network has been constructed only based on known side effects of FDA approved drugs. A drawback is the sole focus on negative clinical parameters, limitation to FDA approved drugs and the neglection of dynamical aspects of drug effects. In^27^ in addition to gene expressin data also information about chemical structures and drug responses have been used. Unfortuantely, the number of drugs for which all three sources of data are available is very limited. A common shortcoming of all these studies is a lack of conceptual explanations of the drug networks.

The ultimate goal in pharmacology is to know all properties, effects and actions of all drugs and componds^28^. Hypothetically, this information could be obtained from clinical trials testing each compound for every existing disease including subtypes and stages. From this information one could measure the similarity between different compounds, e.g., based on clinically relevant parameters. This would give the network structure of an ideal compound-space giving all relationships among all compounds corresponding to an ideal drug association network (iDAN). Due to the practical impossibility of such an approach the question is, is it possible by using genomics data to approximate such an iDAN?

**Figure 1:**
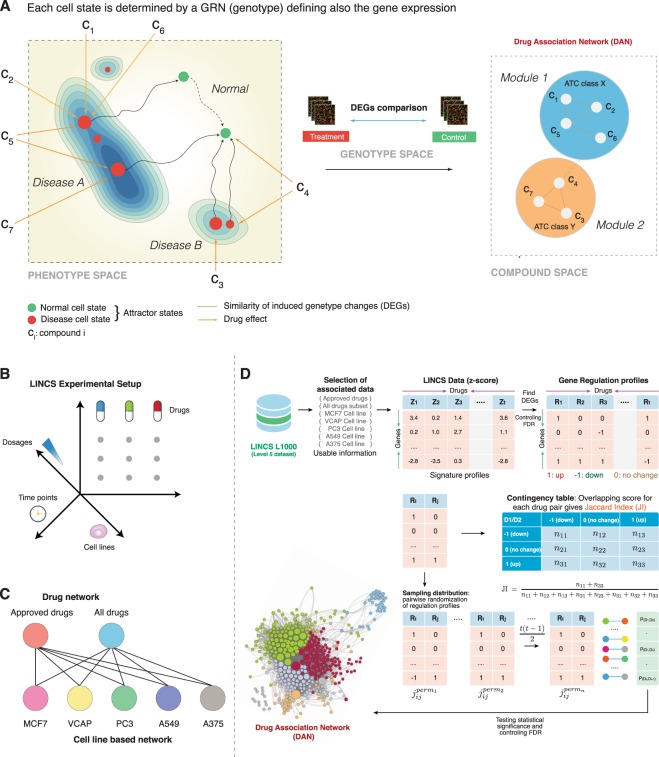
(**A**) Conceptual connection between genotype space, phenotype space and compound space containing DANs. (**B**) Multifacturial experimental space of the LINCS data. (**C**) For our analysis we study 7 different DANs. (**D**) Overview of the connstruction of a DAN. The figure shows the gene expression profile signature of drugs and small molecule compounds from LINCS L1000 subset. Representation of the use of drug-feature matrices of different types to calculate drug connections using Jaccard Index (JI).

The main purpose of our paper is to introduce a computational method that provides such an approximation leading to a systematic organization for the thousands of drugs and small compounds that are available from the LINCS repository. Specifically, we introduce a method for constructing Drug Association Networks (DAN) based on almost two million gene expression profiles for over 20,000 chemical perturbagens and seventy-two human cell lines. In these networks nodes correspond to drugs and two drugs are connected if their profile responses are similar, as measured by the statistical significance of the Jaccard Index (JI). The profile responses for each drug correspond to estimates of “consensus” signature profiles summarizing the transcriptional effect of drugs across multiple treatments on different cell lines and/or different dosages and time points. Overall, the DANs provide a systematic summary of the entire LINCS data repository and the complex pharmacogenomic landscape of drug similarities. For a conceptual overview see Fig. 1A.

For obtaining pharmacogenomically meaningful networks, we construct different DANs based on data from different conditions. Specifically, we construct for each cell line a DAN using only the corresponding drug signature profiles. Furthermore, we construct one DAN limited to FDA approved drugs and one DAN for all drugs and small compounds (comprising FDA approved and non-approved drugs). This leads to condition-specific DANs (see Fig. 1C for their dependencies). In total, we are inferring 74 different DANs.

In order to analyze and interpret the DANs, we investigate the DANs on three different levels. First, we study the structure of the DANs by identifying network modules, also called communities^29–31^. This will allow us to gain insights into the structural properties of the networks. Second, we study drugs pairwise by identifying the presence of significant Anatomical Therapeutic Chemical (ATC) classes in the entire network. This analysis step will show that drugs with similar ATC classes are actually identified in compound space. Third, we study the enrichment of the network modules with respect to ATC classes. By using the ATC classification of drugs, we will demonstrate that the DANs represent a pharmacogenomic landscape of drugs summarizing the entire LINCS repository on a genomic scale.

As a general results, we will show that the ATC code enriched modules in the DANs can be seen as therapeutic attractors of drug classes. We will see that this allows a conceptual extension of the idea of *cancer attractors*^32^ introduced for gene regulatory networks to represent cell states^33,34^ to DANs representing pharmacological states (need name).

Furthermore, in order to communicate the wealth of our obtained results efficiently, we developed a web interface accessible at (http://dan-network.herokuapp.com). Our web application allows to access the drug-drug interactions inferred by our method, and connecting to external links. The features of our DAN user interface enable searching, browsing, exploration and downloading of the network visualizations.

The paper is organized as follows. In the next section we present the Materials and Methods used for our analysis. Then we present our Results and a Discussion. This paper finishes with Conclusions.

## Results

In the following, we first construct DANs from different information corresponding to different characteristics of the LINCS data. This results in DANs having a context specific meaning. Then we will analyze the DANs on three different levels. First, we focus on the structure of the DANs identifying modules in the networks. Second, we study drugs pairwise by identifying the presence of significant ATC classes in the entire network. Third, we study the enrichment of the network modules with respect to ATC classes.

### Construction of drug association networks

**Figure 2:**
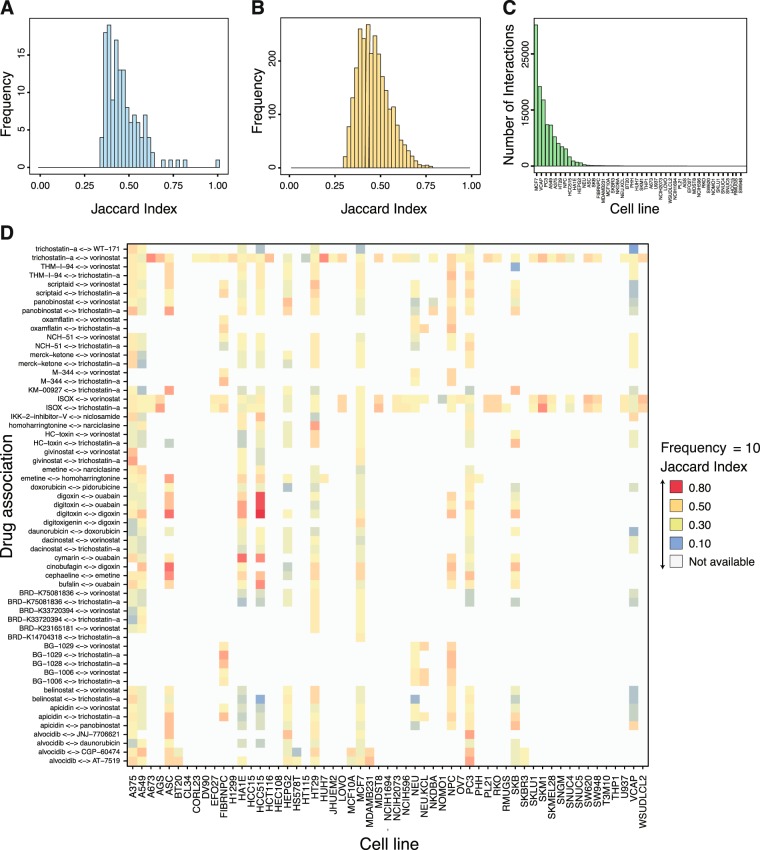
Similarity distribution of drugs over different experiments. (**A**) Distribution of JI of all significant interactions for *N*_approved_ from profiles having between 100–150 DEGs. (**B**) Distribution of JI of all significant interactions for *N*_all_ from signature profiles having DEGs between 700–800. (**C**) Number of significant interactions between drugs for different cell lines. (**D**) Heat map showing drug similarities using JI for selected drug-pairs (y-axis) in dependence on cell lines (x-axis) having a JI larger than 0.5 and appearing in ten or more experiments. The color indicates the value of the JI for drug-pairs. The grey color shows drug-pair not available in a given cell line.

The first network, we construct for FDA-approved drugs with assigned annotations in DrugBank^35,36^. For this reason we call this network *N*_approved_. In total, there are 1139 approved drugs in LINCS, however, only 381 have an ATC annotation. The drugs with DrugBank IDs are repeated in multiple experiments; therefore, the *landmark* genes have multiple z-scores from different experiments. We first average the z-scores for each drug from different experiments and use the consensus of the z-scores to construct the DAN, as described in the method section. From this analysis, we obtain a network with 381 nodes and 4251 significant interactions. From this network, we extract the giant connected component (GCC) having 367 drugs (nodes) and 4244 interactions (edges). In Fig. 2A, we show the distribution of JI of all significant interactions for this network from profiles having between 100 to 150 DEGs.

The second network we construct, we call *N*_all_, is for all available drugs. In LINCS data there are in total 2505 different drugs applied in the different experiments (cell line, dosage and time point). For these, we construct a network with 2505 drugs and 86,585 significant interactions. From this network, we extract the GCC having 2451 nodes and 22636 interactions. In Fig. 2B, we show the distribution of JI of all significant interactions for this network from profiles having between 700 to 800 DEGs. The higher the value of the JI the more genes are commonly up- or down-regulated between two drugs.

Next, we construct 72 networks that are specific for the 72 cell lines. All of these networks are sub-graphs of *N*_all_, i.e., ${N}_{{\rm{all}}}^{C{L}_{l}}\subset {N}_{{\rm{all}}}$, with *CL* = {*list of cell lines in LINCS*}, due to the way we summarize all configurations, see Eqn. 5. In addition, it holds ${N}_{{\rm{all}}}={\cup }_{C{L}_{l}\in CL}{N}_{{\rm{all}}}^{C{L}_{l}}$. That means, *N*_all_ contains all significant interactions identified for any cell line.

For our further analysis, we select from these 72 networks the five networks having the highest number of interactions between the drugs; see Fig. 2C for the frequency distribution of interactions for all cell lines. These cell lines are {*MCF*7, *VCAP*, *PC*3, *A*549, *A*375}. These 5 networks contain the most information, assuming interactions provide informative knowledge. The high number of interactions in each of these networks (more than 10,000) ensures also that a sensible identification of modules is feasible.

In Table 4, we show a summary of these seven networks and their number of nodes and edges. All of these networks correspond to the GCC of the corresponding network. In the following, we will limit our analysis to these seven networks.

### Modules in Dans

Our first analysis consists in the identification of the modules in the seven different DAN networks. For this, we are using a multilevel community module detection algorithm^37^ to find the modules in the networks. The modularity and the number of modules for each network are summarized in Table 4. We would like to remark that the number of the modules correspond to labels, i.e., the same label for different networks does not mean it should contain the same drugs. In general, we find the modularity to be similar among the different networks except for *N*_approved_ and *N*_all_ which is smaller. This is understandable considering the used data for these networks is different to the others. For the number of modules we observe similar values ranging from 11 to 25 modules.

**Figure 3:**
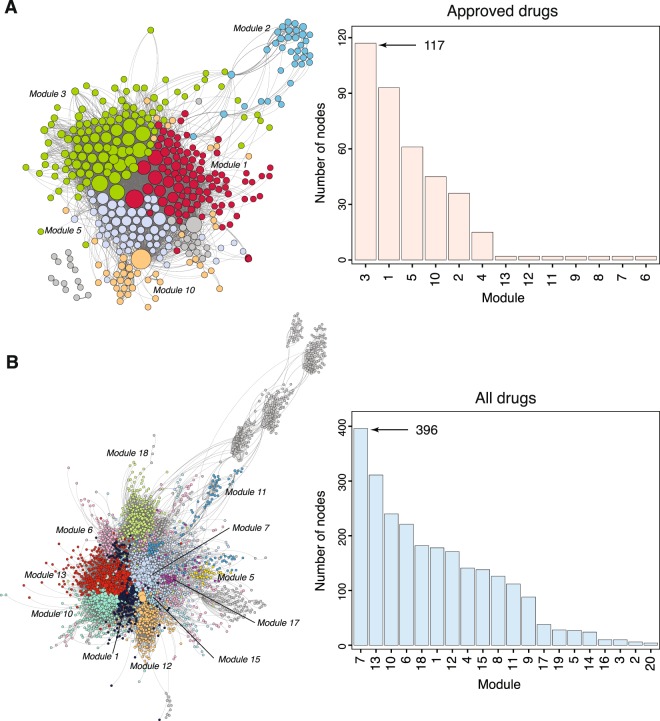
Drug network connecting the most associative drugs using JI and module annotation from LINCS L1000 dataset. The network representation displays drugs as circles (nodes) connected with edges. The colour of drug corresponds to their associative grouped module. (**A**) Shows the network of FDA-Approved drugs with their corresponding module annotations (Left), and the number of nodes in each module of *N*_approved_ (Right) (**B**) The network show All Drugs including approved and non-approved drugs colored based on grouped module (Left), and the number of drugs in each cluster for *N*_all_ (Right).

In Fig. 3, we show the networks for *N*_approved_ and *N*_all_ and the distribution of the number of drugs in the modules. The networks for the 5 cell lines are shown in Fig. 1–5 in the Supplementary File. From the barcharts of boths networks one can see that there are a few modules containing a large number of drugs and the remaining modules contain only a few drugs. These large modules are also clearly visible in the network representation of the DANs on the left-hand-side in Fig. 3. In general, the modules in *N*_all_ are larger than in *N*_approved_ which is understandable because the former DAN contains 2451 nodes whereas the latter has only 367 (see Table 4).

### Significance of ATC interactions in the entire network

**Figure 4:**
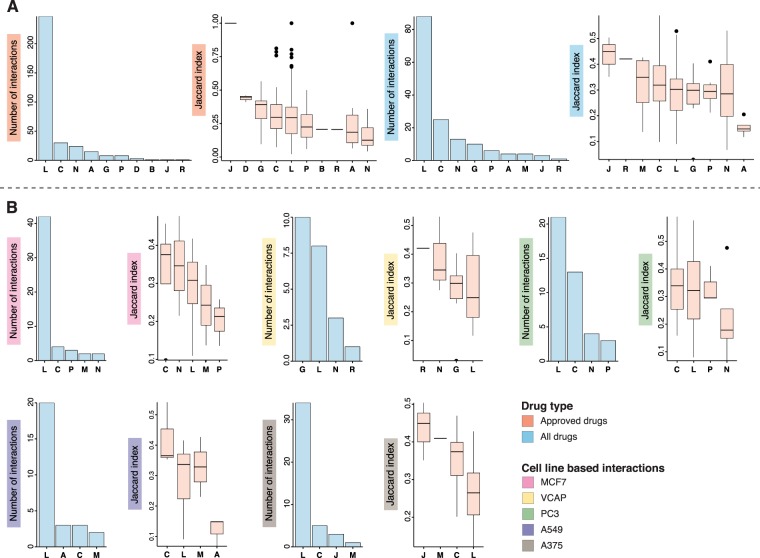
Significant interactions between drugs with the same ATC classes. Here the notation, e.g., *L* means *L* − *L* (x-axis) (similar for other ATC codes) and their corresponding Jaccard Indices. (**A**) Number of significant interactions between the same *ATC* codes (i.e two drugs with the same ATC class) for the networks *N*_approved_ (right) and *N*_all_ (left). The boxplots show the distribution of JI of all significant interactions of drugs which are annotated with the same ATC codes of *N*_approved_ (right) and *N*_all_ (left). (**B**) Results for the 5 networks *N*_MCF7_, *N*_VCAP_, *N*_PC3_, *N*_A549_ and *N*_A375_. Shown results are similar as for (**A**). The colored y-axis label indicate the type of network analysed.

Next, we analyze pairwise interactions between drugs in terms of their corresponding ATC classes. For this analysis, we use all the significant interactions which are annotated with *ATC* codes in the 7 DANs. The number of interactions and the distribution of their JI values are shown in Fig. 4. In this figure, we show only drug pairs beloning to the same ATC class corresponding to homogene interactions, i.e., the label L refers to the interaction of two drugs, both from ATC class L.

For the network *N*_approved_ the number of interactions and their JI values are shown in Fig. 4A (left with red label). One can see that interactions between drugs from the ATC class L occur far more often than for any other ATC class. Interestingly, the differences in the values of the JI for these interactions (shown in the boxplot in Fig. 4A) are not that different for different ATC classes. The results are similar for *N*_all_.

For the other five networks of the cell lines, the frequency of drug annotations and the distribution of JI values are shown in Fig. 4B. From comparing these five networks we make five observations. First, the number of ATC classes is much smaller than for the two networks *N*_approved_ and *N*_all_. Second, the ATC class L is present in all networks for the cell lines. Third, the overlap between the five cell line networks with respect to the ATC classes is smaller than for the two generic networks. Fourth, the network *N*_VCAP_ is the only one having more interactions for the ATC class G. Also the difference between the top 4 ATC classes is smaller than for the other networks, except *N*_PC3_. Fifth, all of the networks share that the ATC class of the larges JI values do not correspond to the ATC class for the largest number of interactions.

In order to reveal robust interaction patterns, we randomize the ATC class labels of the drugs and determine statistically significant ATC interactions classes. For this analysis, we study homogeneous as well as heterogeneous interactions (between drugs from different ATC classes) corresponding to the inter-class effect of drugs. Specifically, we obtain the counts of ATC code combinations from each network (i.e. *A* − *A*, *A* − *C*, *B* − *L* etc.) by counting their occurancy in each DAN. Then we randomise each network 10,000 times to obtain the null distribution for each ATC class combination using the counts of ATC classes as test statistic for each ATC class. From comparing the null distributions with the test statistics we obtaine p-values to which we apply a Bonferroni multiple testing correction to get the adjusted p-values.

These results demonstrate that the inferred network structure of all DANs capturing meaningful drug-specific information that could be revealed by the significance of selected ATC classes.

### Enrichment analysis of network modules

Finally, in order to obtain a pharmacogenomically meaningful interpretation of the DANs, we perform an enrichment analysis of the modules identified in the previous section.

The constructed DANs have nodes corresponding to known and unknown drugs and some of the nodes (drugs) in these networks have Anatomical Therapeutic Chemical (ATC) annotations^38^. We categorized these drugs/nodes with ATC annotations into 14 classes, summarized in Table 2. In addition, we use the label ‘X’ to indicate drugs for which no drug annotation is known.

**Table 1:** Contingency table summarizing the gene regulation profiles *R*_*i*_ and *R*_*j*_ treated by drug *D*_*k*_ and *D*_*l*_.

*D*_*i*_↓/*D*_*j*_→	−1 (down)	0 (no change)	1 (up)
−1 (down)	*n*_11_	*n*_12_	*n*_13_
(no change)	*n*_21_	*n*_22_	*n*_23_
(up)	*n*_31_	*n*_32_	*n*_33_

Here *n*_*kl*_ are integer numbers giving the common genes in the categories *k*, *l* ∈ {*up*, *nochange*, *down*}.

**Table 2:** Description of ATC annotations.

Code	Description
A	Alimentary tract and metabolism
B	Blood and blood forming organs
C	Cardiovascular system
D	Dermatologicals
G	Genito urinary system and sex hormones
H	Systemic hormonal preparations, excl. sex hormones and insulins
J	Antiinfectives for systemic use
L	Antineoplastic and immunomodulating agents
M	Musculo-skeletal system
N	Nervous system
P	Antiparasitic products, insecticides, and repellents
R	Respiratory system
S	Sensory organs
V	Various

The first level of the ATC classification represents the organ or system in the body on which the therapeutic effect.

**Figure 5:**
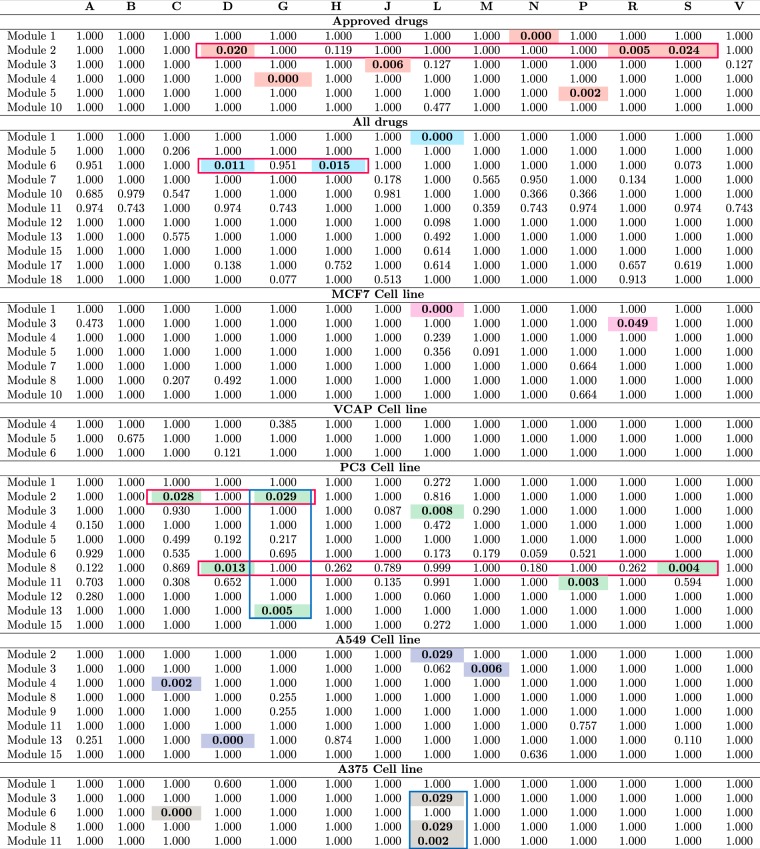
Enrichment of individual modules in the DANs. Shown are the BH corrected q-values of Fisher’s exact tests for the enrichment of ATC codes in each of the modules of the DANs. Modules not shown, do not contain any enriched ATC code. The highlighted cells are statistically significant. The horizontal and vertical boxes highlight the multiple occurance of ATC classes in modules and multiple enriched modules for an ATC class respectively.

We performed an enrichment analysis of drugs with ATC codes for the modules detected in each network. In order to test the statistical significance of ATC classes, we use Fisher’s Exact Test^39^. Since we are testing multiple hypothesis tests for each module, we apply a Benjamini Hochberg correction to control the FDR. In the enrichment analysis we first find the total number of drugs in a module which are labelled with ATC codes and then we performed Fisher’s Exact test to determine which ATC labels are overrepresented in a particular module. The results of this enrichment analysis are shown in Fig. 5.

In *N*_approved_, the N (Nervous system) group is overrepresented in first module. The ATC groups R (Respiratory system), S (Sensory organs) and D (Dermatologicals) are enriched to the second module. The ATC group J (Antiinfectives for systemetic use), G (Genito-urinary system and sex hormones) and P (Antiparasitic products, insecticides and repellents) are enriched in 3, 4 and 5 modules. This is interesting to highlight, since the drugs which are overrepresented in the same modules of different classes perturb common genes or a similar subset of genes. This information can be used for further investigation to see if those drugs can perturb common pathways.

In the network (*N*_all_), the ATC group L (Antineoplastic and immunomodulating agents) is overrepresented in first module. ATC groups H (Systemic hormonal preparations, excluding sex hormones and insulins) and D (Dermatologicals) are enriched to the sixth module, however group S (Sensory organs) also show a low q-value (0.073, which is not significant).

For the network *N*_MCF7_, it shows the ATC group L (Antineoplastic and immunomodulating agents) and R (Respiratory system) are enriched in the first and third modules. However, the ATC group M show a low q-value (0.090) in module 5.

For the network *N*_VCAP_, no ATC group is enriched in any module however, ATC group *D* (Dermatologicals) show a low q-value (0.121) in module 6.

In the network *N*_PC3_, the ATC groups G (Genito-urinary system and sex hormones) and C (Cardiovascular system) are enriched in module 2. The ATC group L (Antineoplastic and immunomodulating agents), in module 3, also ATC group J (Antiinfectives for systemic use) has a low q-value (0.087) in module 3. The ATC group N (Nervous system) shows a low q-score (0.059) in module 6. The ATC groups S (Sensory organs) and D (Dermatologicals) are enriched in module 8. The ATC group P (Antiparasitic products, insecticides and repellents) is also enriched in module 11. The ATC group L (Antineoplastic and immunomodulating agents) show a low q-score (0.06) in module 12. The ATC group G (Genito-urinary system and sex hormones) is enriched in module 13.

In the network *N*_A549_, the ATC group L (Antineoplastic and immunomodulating agents) is enriched in module 2. The ATC group M is enriched in module 3, ATC group C is enriched in module 4. However, The ATC group L (0.062) and S (0.11) show low q-values in modules 3 and 13 respectively.

In The network *N*_A375_, the ATC group L (Antineoplastic and immunomodulating agents) is enriched in modules 3, 8 and 11 respectively. The ATC group C (Cardiovascular system) is enriched in mdoule 6.

The summary of the enrichment analysis of the ATC groups for the modules of the different networks is shown in Table 5. In this table, we highlighted the ATC groups which are enriched in at least one module in different networks. We also include those ATC groups which are not significant but holds low q-values between 0.05 < *α* < 0.15.

### Web interface for DAN of drugs

**Figure 6:**
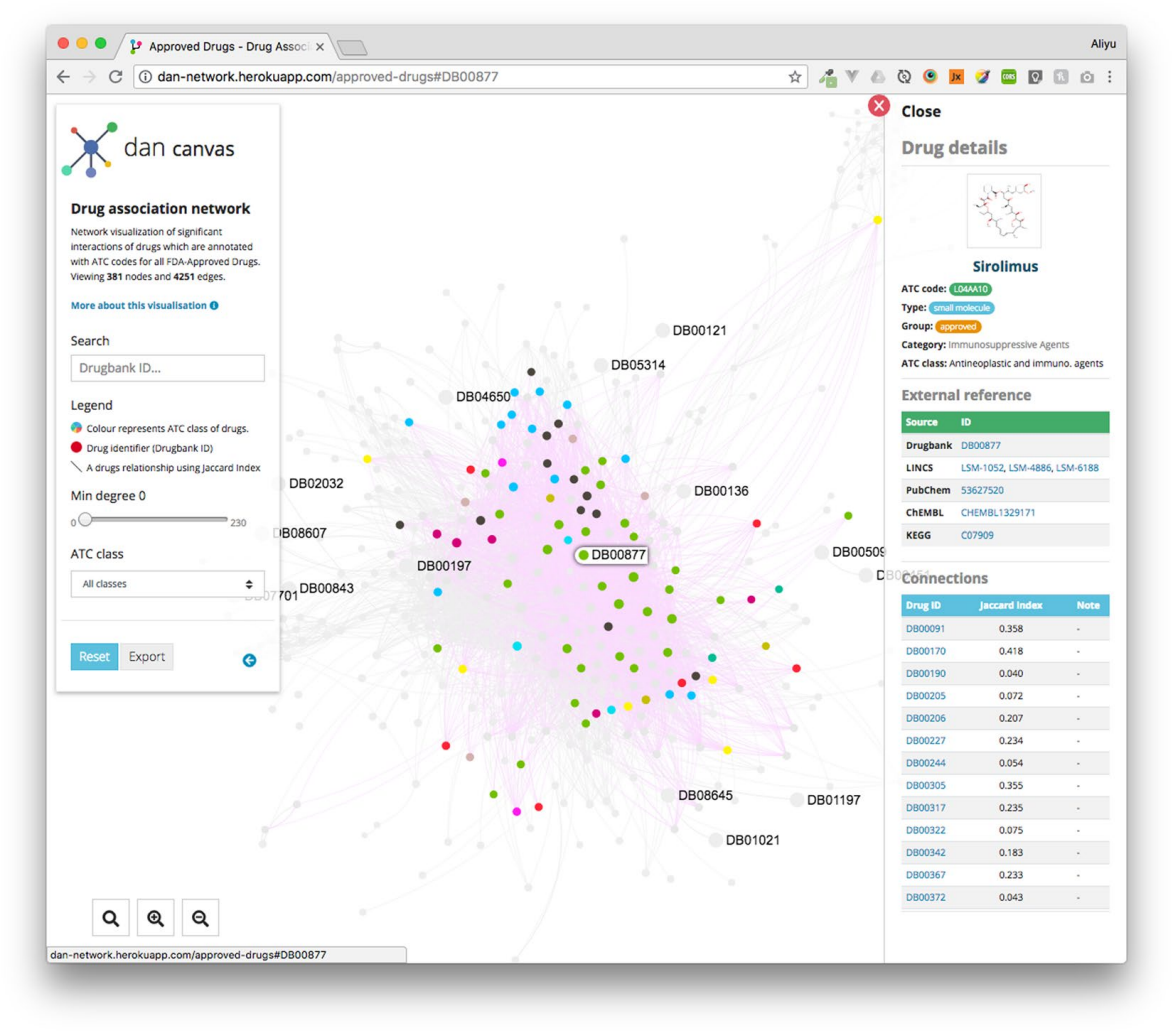
The website view of the DAN network. This website shows our results of the drug-drug interaction network for all 20,009 drugs and small-molecule compounds profiled in the LINCS L1000 signature gene expression profiles.

Due to complexity of our results making it difficult to communicate all details, we developed an interactive web application. The web application is publicly available at http://dan-network.herokuapp.com/ showing visualizations of all 7 DANs summarized in Table 4. For the technical realization for the visualization of the networks we developed our web interface using the NodeJs^40^ and SigmaJS^41^ libraries. Each node in the network (drug) has a dedicated pane with a list of the relevant associations and external resources to websites such as: DrugBank, PubChem, LINCS Portal, ChemBL and KEGG Ligand with relevant identifiers. That means, a user can interactively explore the interactions in all 7 DANs obtaining pharmacological information from the linked data resources. A screen shot of our web application is shown in Fig. 6.

## Discussion

In our paper, we based our analysis on the LINCS data repository providing compreshensive information about the effect of drugs or compounds on gene expression changes. This means LINCS enables an estimation of the linkage between genotype, phenotype and therapies and to identify key genes which are a significant part of the biological processes related to phenotype differences as approximated by gene expression values.

For our study, we went beyond single genes because we were aiming at a comprehensive overview of the systems relations among all drugs tested in LINCS. In order to accomplish this, we utilized differentially expression profiles to estimate DANs. Specifically, our analysis started by constructing DANs to estimate the similarity between drug pairs using the Jaccard Index, which estimates the proportion of differentially expressed genes that are common in the corresponding expression profiles. If two drugs showed a statistically significant similarity, we connected them by an edge. In this way, we constructed 7 different DANs for 7 different conditions, which we further analyzed. The results of these networks are summarized in Table 4.

We analyzed the DANs on three differnt levels. First we studied the structure of the DANs by identifying network modules. Second, we studied the drugs pairwise by identifying the presence of significant ATC classes in the entire network. Third, we studied the enrichment of the network modules with respect to ATC classes.

The significant pairs in the networks show a variable JI distribution, shown in Fig. 2A,B. In general, the effect of drugs in terms of differentially expressed genes varies, i.e., some drugs show a strong effect, which means a large number of differentially expressed genes, while other drugs have a moderate effect changing the expression of only a small number of genes. If a drug, *D*_*i*_ has a moderate effect, i.e., a small number of differentially expressed genes, but a strong overlap with the drug, *D*_*j*_, which has a strong effect on the genes, i.e., it causes a larger number of differentially genes, the JI will be significant but not high. In such cases the interaction may not describe the same functionality of both drugs, but it can have a similar effect on some subset of gene targets. On the other hand, if two drugs have a similar proportion of differentially expressed genes and overlap strongly then the corresponding JI is higher.

After the construction of the networks, we identified modules in the networks. For this we employed the multilevel community algorithm^37^. The results of this analysis are summarized in Table 4. In general, the modularity of the networks for the five cell lines is higher than for *N*_all_ and *N*_approved_, which has the lowest modularity. For the number of identified modules this distinction is no longer present. It is interesting to note that the number of modules in all networks is of the same order of magnitude as the number of our ATC classes (which is 14).

It is interesting that the modularity of *N*_all_ and *N*_approved_ is different to the five cell line DANs because these two network types are indeed quite different from each other due to the different information used for their construction.

These results suggest that the modules in the networks could represent drugs or drug classes effecting similar targets. That means drugs in the same module have a similar effect on some common gene targets, because of their significant overlapping of differentially expressed genes as measured by the JI. This can also be interpreted as follows: The presence of drugs in different modules suggests that each module can identify a different type of target-set, which is independent from other target-sets for different drugs. For instance, for *N*_approved_, we identify 13 modules which means that there are 13 distinct effect types of drugs. Interestingly, this number is very close the total number of ATC classes we were using, which is 14 (see Table 2).

In order to test this idea further, we performed an enrichment analysis of the network modules testing for the enrichment of ATC classes. The results are summarized in Fig. 5. Due to the complexity of these results, we discuss them in three steps. First, we discuss results for all networks combined. Second, we discuss network specific characteristics of significant modules and ATC classes. Third, we discuss networks and modules indivdually to identify commonalities.

First, from our results (see Table 5) we see that the total number of significant modules (SM (all networks)) for all networks enriched for the ATC classes is low varying between 7 (for ATC class L) and 0 (for ATC class A, B and V). Most ATC classes are only enriched in 1 or 2 modules in all networks, e.g., ATC class H, J, M, N, P, R and S.

Second, when looking at the networks individually, we found that the total number of enriched modules (SM) per network varies between 5 (for *N*_approved_) and 0 (for *N*_VCAP_). Similarly, the number of significant ATC classes (SC) per network varies between 7 (for *N*_approved_) and 0 (for *N*_VCAP_), see Table 5. Taken together, these observations confirm our interpretation of the findings for the number of modules, which did not consider ATC enrichments, underlining the representative character of the modules for ATC classes.

Third, we are looking at networks and modules indivdually. From these we can obtain the following summary for this level. Overall, we can identify four different types of drug-module enrichments discussed in the following.

### Single-drug class in individual modules

For this type of enrichment, we find only one enriched ATC class per module in a DAN. That means there is an unique relation between an ATC class and a module in a network. From our results, we find that the *N*_approved_ and *N*_A549_ have four modules which are enriched for a single ATC class, *N*_MCF7_ and *N*_PC3_ have two such modules, *N*_all_ and *N*_A375_ have one module, and *N*_VCAP_ has no significant module.

The interpretation for these results is that each module is characteristic for a set of drugs represented by an ATC code and could be used to predict the function of unknown drugs within this module because they are likely to have common targets. This could be used to predict the function of unknown drugs or drug repositining.

### Single-drug class in multiple modules

For this type, an ATC class is enriched in more than one module. For instance, ATC class L is enriched in 3 modules in *N*_A375_; see the vertical boxes in Fig. 5. Furthermore, ATC class G is enriched in two modules in *N*_PC3_. This suggests that drug class G and L have possibly three, respectively two independent target-sets effected by these drugs. This means ATC classes G and L have multiple target sets which are at least partially independent from each other.

The interpretation is that if in a network a single ATC class is enriched in multiple modules, the drugs from this ATC class are heterogenously separated targeting different subsets of genes.

### Multiple-drug classes in a single module

For this type, we find more than one ATC class enriched in a module. The *N*_approved_ network has three ATC classes (D, R, and S) enriched in module 2; see the horizontal boxes in Fig. 5. The netwok *N*_PC3_ has two modules enriched with two drugs. Specifically, module 2 is enriched by ATC class C and G and moduel 8 is enriched by ATC class D and S. Finally, *N*_all_ has module 6 enriched by ATC class D and H.

Our interpretation for this is if multiple ATC classes are enriched in a single module, this means that, e.g., two drugs from two different ATC classes have at least partially common targets. These targets hight be higher order, i.e., not directly targeted by a drug but further downstream, but enough to change the differential expression of such genes. This could be used to predict a drug repurposing.

### Multiple Drug classes in multiple modules

For this type, we find an ATC class enriched in multiple modules together with further enriched ATC classes; see the intersection of a horizontal and vertical boxe in Fig. 5. For this type, we find merely one network *N*_PC3_ whereas ATC class G is enriched in module 2 and 13 and the enrichment in module 2 is shared with ATC class C.

This result indicates that a drug class has multiple independent target-sets and could be used for predicting the repurposing of known drugs as well as predicting the function of unknown drugs.

Combining all our findings, our results have a similarity to the conceptual idea of *cancer attractors* introduced by^32,42^ and, e.g., studied in^33,34^. The authors analyzed gene regulatory networks and showed that cell types can be seen as attractors in the epigenetic landscape representing the phenotype space of an organism, see Fig. 1A. That means the developmental state of cells giving raise to different cell fates can be seen as dynamical gene networks chaning their structure over time and as a consequence changing their position in the epegenetic landscape. Similar studies have been conducted by^43–45^. In^33^ it has been argued that cancer cells are trapped in abnormal attractors allowing in this way the extension of the conceptual idea of *attractors* in gene regulatory networks to general abnormal or tumor cell types in diseaes beyond cancer^46–48^.

Our study adds in a non-trivial way to this because we do not study gene regulatory networks but DANs, where the drugs/compounds correspond to the nodes of the network instead of genes. Due to the fact that we determine the similarity between pairs of drugs based on hundreds or even thousands of expression profiles, for certain conditions, a DAN integrates dozens of individual gene regulatory networks, each representing a particular cell state, see Fig. 1A. This includes a temporal integration of the cells due to the perturbation effect to the exposed drugs. This means that despite the fact that the DANs are static they nevertheless represent dynamical states of the underlying cells. Hence, a DAN is capable of representing many different states of cells, corresponding to phenotypes, simultenously and allows the integrated representation of the drug landscape.

It is important to emphasize the difference between the different ‘spaces’ considered. GRNs are embedded into the genotype space describing the activity of genes, whereas the epigenetic landscape, representing the phenotype space, describes cell states and their transitions. Here a cell state can correspond to a normal cell type or an abnormal tumour or disease cells. These states are the *attractors* of  ^32,42^. Each cell state has a corresponding GRN and, hence, a projection into genotype space. Our DANs are embedded into the compound space representing therapeutic interventions. Each state in the compound space corresponds to a drug/compound that is connected to the phenotype space to abnormal and normal cell states. The connection between these three spaces is visualized in Fig. 1A.

For our DANs, we found a graph-theoretical correspondence of an ‘attractor’ state in phenotype space, by the modules in the networks in the compound space. This could be demonstrated by utilizing information about the ATC classification of known drugs. In this way we complemented LINCS with information from DrugBank about known effects of drugs.

For enabling an efficient exploration and reusage of our results, we developed an interactive web interface that can be used to view, explore, and link drug associations for our results. The interface also provides an integration with external resources via added links, curated mappings, and external IDs. Content from other resources such as PubChem has been incorporated into the DAN web interface enabling End users to view information and explore new hypotheses of drug associations. These features could facilitate further research in the field on a large-scale and in addition could provide health care professionals with a valuable systems pharmacogenomics source.

Finally, we would like to note that it appears desirable to integrate different types of genomics data, e.g., transcriptomics, proteomics and metabolomics data, to establish in this way an integrated systems pharmacogenomics landscape of drug similarities. Unfortunately, the LINCS database, on which our analysis is based, nor any other current database, does not provide those different types of data that would allow to realize this approach practically. For this reason, our approach is the most feasible one considering the current practical data constraints and can be as an approximation of thereof. On a more theoretical note, we would like to add that even if one could realize an integrated systems pharmacogenomics landscape it is unclear if all different genomics data types are actually required or if they are, at least partially, redundant. Only future studies can shed light on this conceptual issue.

## Conclusion

In this paper, we developed a systems pharmacogenomics approach and applied it to data from the LINCS repository. As a result, we constructed *Drug Association Networks* summarizing hundreds of drugs and thousands of compounds systematically with respect to their therapeutic effects. We showed that the modular structure of the DANs represent enriched ATC classes thus integrating the drug induced changes on the genotype states of the cells.

## Materials and Methods

### Drug perturbation data from LINCS data

The LINCS L1000 data comprises of 5806 genetic perturbations (e.g., single gene knockdown and over-expression) and 16,425 perturbations induced by chemical compounds (e.g., drugs)^49^. About 1.3 million gene expression have been profiled and collected for this project using the L1000 technology^50^. The L1000 platform has been developed at the Broad Institute by the connectivity map (CMap) team to facilitate rapid, flexible and high throughput gene expression profiling at a lower cost. However, the L1000 technology only measures expression for 978 *landmark* genes and the expression values for the rest of the transcriptome are estimated using a computational model based on Gene Expression Omnibus (GEO)^51^ data. In this paper, we used the level 5 signature data of drug perturbations in various cell lines. Overall, the LINCS data were generated from a multifacturial experimental space, see Fig. 1B.

### DrugBank database

DrugBank is a comprehensive drug data resource that contains records about chemical, pharmacological, and pharmaceutical features of more than 8,000 drugs, including the 2016 FDA-approved drugs^52^. We used version 5.0.11 (released 2017-12-20) of the DrugBank database for our analysis. To make the cross-platform comparisons compatible, we considered the DrugBank ID as the identifier of drugs across the DrugBank and LINCS databases. For our analysis, we used the Anatomical Therapeutic Chemical (ATC) classification codes, controled by the WHO, shown in Table 2. This classification categorizes drugs into different groups/classes according to the organ or system on which they act, their therapeutic effect, and their chemical characteristics. For our analysis we use the first ATC level, which gives 14 main anatomical classes.

### Metadata pipeline

The LINCS data API provides a programmatic pipeline to annotations and perturbational signatures in the L1000 dataset via a collection of HTTP-based RESTful web services. An example of these services includes; Cell Service, which is a service that describes the cell line meta-information. The API services provided by the LINCS API for querying the L1000 metadata support complex queries via simple HTTP GET requests that can be executed in a web browser or most programming languages such as R and Python.

### Transcriptional profiles and small molecules diversity

We downloaded the L1000 raw z-score vectors from the GEO repository and pre-processed them using the R L1000 tools^53^. A signature of a small molecule is defined as a vector of z-score values, representing the differential expression between samples treated with small molecules and control samples. That means a z-score signature summarizes the effect of the treatment with a small molecule. This is in depencence on experimental condition, e.g., dosage, time point, cell line etc.

In total, there are 169, 239 z-score signature profiles marked with the highest signature count that satisfied the well- and plate-based quality control. This signature profile subset covers 20, 009 small molecules (out of 49, 400 perturbagens) that were repeatedly measured with 1 to 8 replicates. For our analysis, we select the time points 6, 24 and 48 *h* because they represent by far the majority of conditions. From this we find in total 158, 054 signature profiles (i.e., any combination of the small molecule, time, and cell line) we use for our analysis. In Table 3, we show some summary statistis of this data set.

**Table 3:** Summary of z-score signature profiles for DEGs between treatments and controls on the cell line subset.

	Signature profile	Small molecule
No significant gene	24	19
At least 1 significant gene	158,030	19,957
At least 50 significant genes	58,739	15,714
At least 100 significant genes	23,867	8,211
**Total**	**158,054**	**20,009**

**Table 4:** Summary of seven DANs constructed from different information.

DAN	Used information	Drugs	Edges	Modularity	No. of Modules
*N*_approved_	Approved drugs	367	4244	0.318	13
*N*_all_	All drugs	2451	22636	0.554	20
gray *N*_MCF7_	MCF7 cell line	750	7144	0.623	11
*N*_VCAP_	VCAP cell line	520	2727	0.749	25
*N*_PC3_	PC3 cell line	612	4314	0.644	17
*N*_A549_	A549 cell line	380	2122	0.561	22
*N*_A375_	A375 cell line	635	4286	0.636	14

Shown is the information of the giant connected component. Column two describes the used information that characterizes the underlying data for each network.

**Table 5:** Summary of module enrichments shown in Table 5 for all DANs.

DAN/ATC code	C	D	G	H	J	L	M	N	P	R	S	SC	SM
Approved drugs		1	1		1			1	1	1	1	7	5
All drugs		1		1		1						3	2
gray MCF7 cell line						1				1		2	2
VCAP cell line												0	0
PC3 cell line	1	1	2			1			1		1	6	5
A549 cell line	1	1				1	1					4	4
A375 cell line	1					3						2	4
**SM (all networks)**	3	4	3	1	1	7	1	1	2	2	2		

The columns show ATC classes highlighting if ATC codes are enriched in at least one module in the entire network (see Table 5). SC gives the number of significant ATC classes and SM gives the number of significant modules per network. SM (all networks) gives the number of significant modules in all DANs.

The z-score signature vectors were used to study the effect of a drug treatment on the differential expression of genes. We used the threshold >2.0 to indicate upregulation and <−2.0 to indicate down-regulation of a gene respectively.

### Mapping small molecules to external databases

The L1000 small molecules were assayed across multiple cell lines, experimental replicates, dosages and time points. For this reason, we mapped DrugBank compounds and the directly measured (landmark) genes to calculate a single transcriptional profile across multiple signatures for each L1000 small molecule. We also mapped the L1000 small molecules to external database sources in UniChem database^54^. We achieved this by querying UniChem with the InChIKey of each L1000 compound via UniChem API. This allows us to map the L1000 small molecules not only to DrugBank, but also to PubChem, ChEMBL, and KEGG Ligand covered by UniChem (see Table T1 in Supplementary File 1). The pipeline enables us also to identify FDA-Approved drugs and to map them to the L1000 small molecule identifiers.

After mapping the DrugBank identifiers to small molecules, the identifiers were used to calculate the signature profile consensus for each drug. The purpose for computing consensus is to combine signature profiles for the same perturbation under different conditions (e.g., cell types, different dosages, or time points). The signature profiles consensus were obtained using the following; First, we calculated the Spearman rank correlation of all signatures that belong to a drug identifier in DrugBank. Second, we calculated the weights by taking the mean correlation to normalize the similarities (Total correlation, see Fig. S1 in Supplementary File 1). Third, we multiplied the z-score signatures by their similarity weights. Last, we sum up the weighted z-score vectors to form a single signature consensus.

### Drug association network

The basic idea of the drug association network (DAN) is to generate a network where different drugs show a similar effect on gene expressions which means that the number of genes affected by them has the same type of expression profiles compared to the control data. For example, for a particular cell line treated by drug *D*_*i*_ and *D*_*j*_ having observed phenotype changes ${\hat{P}}_{i}$ and ${\hat{P}}_{j}$, these phenotypes will be similar $({\hat{P}}_{i} \sim {\hat{P}}_{j})$ if the two drugs influence (overexpression or underexpression compare to a control state) similar genes. In order to estimate the similarity between two drugs we use a Jaccard-like index^55^ between two vectors of genes which are characterized as 1 (up), −1 (down) and 0 (no change) by drugs *D*_*i*_ and *D*_*j*_. In the first step, we obtain a matrix by converting the z-scores of drug-treated expression data to a matrix of categorical data-type whereas rows represent genes and drugs correspond to columns. In this matrix, genes are categorized as differentially expressed and non-differentially expressed genes. The differentially expressed genes are labelled by 1, for up-regulated, and −1 for down-regulated. The non-differentially expressed genes are labelled by 0. In the second step, we measure the overlapping score between pairs of drugs by using a JI as described in Eqn. 1. The JI gives a ratio of differentially expressed genes which are common between a pair of drug-treated data w.r.t. all other genes which are differentially expressed in at least one drug-treated data. In the third step, we test the significance of the Jaccard Index. We perform the significance test with a non-parametric approach by randomizing gene labels of each drug data vector independently. This allows us to estimate the sampling distribution of the null hypothesis. A schematic overview for the construction of a DAN is shown in Fig. 1D.

#### Jaccard Index

Let *D*_*k*_ and *D*_*l*_ be two drugs with regulation profiles *R*_*i*_ and *R*_*j*_. *R*_*i*_ and *R*_*j*_ are two vectors of length *n*, whereas *n* is the number of genes. Their components correspond to (I) down-regulation (−1), (II) no-change (0) or (III) up-reguation (1). The Jaccard Index (JI) can be estimated from the contingency table (see Table 1) giving the overlap between the two regulation profiles representing the effect of the drugs *D*_*k*_ and *D*_*l*_:

1 $${J}_{ij}=J({R}_{i},{R}_{j})=\frac{{\Vert {G}_{i}\cap {G}_{j}\Vert }_{/\Vert 0,0\Vert }}{{\Vert {G}_{i}\cup {G}_{j}\Vert }_{/\Vert 0,0\Vert }}=\frac{{n}_{11}+{n}_{33}}{{n}_{t}}$$

here *n*_*t*_ = *n*_11_ + *n*_12_ + *n*_13_ + *n*_21_ + *n*_23_ + *n*_31_ + *n*_32_ + *n*_33_ is the number of genes showing differential expression.

#### Construction of the drug association network

The construction procedure for the DAN consists of 11 steps and is based on z-score vectors available in LINCS. Every z-score vector, *Z* = {*z*_1_, *z*_2_ ..., *z*_*n*_} whereas *n* is the total number of genes, is a function of experimental conditions, including a drug *D*_*k*_ and a cell line *CL*_*m*_, which was exposed to drug *D*_*k*_. For briefity we simply write *Z* = *Z*(*D*_*k*_,*γ*) to indicate that a z-score is a function of drug *D*_*k*_ and further conditions summarized by *γ*. We call (*D*_*k*_,*γ*) a configuration. Due to this dependency, *Z* = *Z*(*D*_*k*_,*γ*) can be seen as a profile for drug *D*_*k*_.

For reasons of notational simplicity, we index the configurations (*D*_*k*_,*γ*) by an integer number. That means we map (*D*_*k*_,*γ*) to *c*_*h*_ ∈ *C* = {*c*_1_, …, *c*_*t*_} = {1, …, *t*}, whereas *t* is the total number of configurations. This leads to the notation

2 $$Z=Z({D}_{k},\,\gamma )=Z({c}_{h})$$

we will use in the following.

1. This step is only used for *N*_approved_: Summarize the z-scores for all configurations with the same drug, i.e., *DC*_*k*_ = {*c*_*i*_, *c*, … *c*_*k*_} whereas every *x* ∈ *DC*_*k*_ contains drug *D*_*k*_. The summarized values are given by
  3 $$Z^{\prime} =\frac{1}{n}\sum _{x\in D{C}_{k}}\,Z(x).$$
  In this case the total number of remaining z-scores corresponds to the number of configurations and the number of drugs. Re-indexing of the configurations gives *c*_*h*_ ∈ *C* = {*c*_1_, …, *c*_*t*_} whereas *t* is now the number of different drugs.
2. Convert every z-score vector into a *p*-value vector, *P* = {*p*_1_, *p*_2_..., *p*_*n*_}, i.e., *P* = *P*(*c*_*h*_).
3. Convert every p-score vector into a q-value vector (controlling FDR with Benjamini and Hochberg (BH) method^56^), *Q* = {*q*_1_, *q*_2_ ..., *q*_*n*_}, i.e., *Q* = *Q*(*c*_*h*_).
4. Construct a matrix *R* of differentially regulated genes for all configurations *c*_*h*_, i.e., *R* is a (*n* × *t*) matrix, whereas the components of this matrix correspond to (I) down-regulation (−1), (II) no-change (0) or (III) up-reguation (1).:
  For each configuration *c*_*h*_, we have the corresponding z-score vector *Z*(*c*_*h*_) and the corresponding *q*-value vector *Q*(*c*_*h*_). The function *f*:(*Z*(*c*_*h*_), *Q*(*c*_*h*_))_*i*_ → *M* maps from the q- and z-value of a gene *i* to its regulation categories, i.e., *M* = {−1, 0, 1}. Specifically, the function *f*(*z*_*i*_(*c*_*h*_), *q*_*i*_(*c*_*h*_)) is defined as follows:
  $$f({z}_{i}({c}_{h}),{q}_{i}({c}_{h}))=\{\begin{array}{ll}-1 & :{q}_{i}({c}_{h})\le \,\alpha \,{\rm{and}}\,{z}_{i}({c}_{h})\, < 0\\ 1 & :{q}_{i}({c}_{h})\le \,\alpha \,{\rm{and}}\,{z}_{i}({c}_{h})\, > 0\\ 0 & :{\rm{otherwise}}\end{array}$$
  This gives $${R}_{i,h}=f({z}_{i}({c}_{h}),{q}_{i}({c}_{h}))$$.
5. Using *R* to calculate the Jaccard index (*J*_*ij*_) as defined in Eqn. 1 for each pair of configurations *c*_*i*_ and *c*_*j*_, with ${c}_{i}\ne {c}_{j}$ and *c*_*i*_, *c*_*j*_ ∈ *C*. Specifically, calculate *J*_*ij*_ = *J*(*R*_*i*_, *R*_*j*_), whereas the *R*_*i*_ and *R*_*j*_ are the columns of matrix *R* for the configurations *c*_*i*_ and *c*_*j*_.
6. Test the significance of a Jaccard Index for each pair of configurations by the following hypothesis.
  *H*_0_: The number of differentially expressed genes overlapping in two dataset treated by drugs *D*_*i*_ and *D*_*j*_ is zero.
  *H*_1_: The number of differentially expressed genes overlapping in two dataset treated by drugs *D*_*i*_ and *D*_*j*_ is not zero.
7. The sampling distribution is obtained from gene-label randomizations for each pair of configuration profiles *R*_*i*_ and *R*_*j*_ from which the corresponding Jaccard index, *J*_*ij*_ = *J*(*R*_*i*_, *R*_*j*_), is determined. This results in the permuted Jaccard indices, ${J}_{perm}(ij)=\{{j}_{ij}^{per{m}_{1}},{j}_{ij}^{per{m}_{2}}\ldots {j}_{ij}^{per{m}_{L}}\}$ for *L* = 2000.
8. From *J*_*perm*_(*ij*), we estimate the p-values by:
  $${p}_{i,j}=Pr({j}_{i,j} > {j}_{i,j}^{perm})=\frac{{\sum }_{k=1}^{L}I({j}_{i,j} > {j}_{i,j}^{per{m}_{k}})}{L}$$
  This gives *P*^*J*^ = {*p*_1,2_, *p*_1,3_, …, *p*_*n*,*n*−1_}, containing in total $\frac{t\cdot (t-1)}{2}$ different p-values.
9. Controling the FDR by BH we convert *P*^*J*^ into *q*-values, *Q*^*J*^ = {*q*_1,2_, *q*_1,3_, …, *q*_*n*,*n*−1_}, consisting in total of $\frac{t\cdot (t-1)}{2}$ different q-values.
10. Construct a matrix *B* for all configurations *C* by using the *q*_*ij*_ values:
  4 $${B}_{{c}_{i},{c}_{j}}=\{\begin{array}{ll}1 & :{q}_{i,j}\le \alpha \\ 0 & :{\rm{otherwise}}\end{array}$$
  Here $${c}_{i},{c}_{j}\in C$$.
11. Construct a DAN by summarizing all configurations with the same drug, i.e., *DC*_*k*_ = {*c*_*i*_, *c*, … *c*_*k*_} whereas every *x* ∈ *DC*_*k*_ contains dug *D*_*k*_

5 $${A}_{{D}_{k},{D}_{l}}={\rm{\Theta }}(\sum _{x\in D{C}_{k},y\in D{C}_{l}}\,{B}_{xy})$$

here Θ(*w*) is the theta function which gives 1 for *w* > 0 and 0 otherwise.

## Supplementary information

LaTeX Supplementary File

Supplementary Dataset 1
