# Supplementary material for: Systems Pharmacogenomic Landscape of Drug Similarities from LINCS data: Drug Association Networks
Source: Sci Rep. 2019 May 24;9:7849. doi: 10.1038/s41598-019-44291-3 (PMC6534546; doi:10.1038/s41598-019-44291-3)
Supplement: Supplementary file 1 — LaTeX Supplementary File [file 41598_2019_44291_MOESM1_ESM.pdf]

Systems Pharmacogenomic Landscape of Drug Similaritiesfrom LINCS Data: Drug Association Networks

Aliyu Musa, Shailesh Tripathi, Matthias Dehmer, Olli Yli-Harja, Stuart A. Kauffman and Frank Emmert-Streib

October 3, 2018

Supplementary table(s)

Table 1: List of five small molecule/compound databases available in UniChem data source.

Source name	Description	% of drugs mapped
PubChem [1]	A database of normalized PubChem compounds (CIDs) from the PubChem Database.	89.34
ChEMBL [2]	A database of bioactive drug-like small molecules and bioactivities abstracted from the scientific literature.	87.47
KEGG Ligand [3]	KEGG LIGAND is a composite database consisting of COMPOUND, GLYCAN, REACTION, RPAIR, RCLASS, and ENZYME databases, whose entries are identified by C, G, R, RP, RC, and EC numbers, respectively.	61.21
LINCS [4]	The LINCS DCIC facilitates and standardized the infor- mation relevant to LINCS assays as described in http://lincsportal.ccs.miami.edu/SmallMolecules/	93.45
DrugBank [5]	A database that combines drug (i.e. chemical, pharmacological and pharmaceutical) data with drug target (i.e. sequence, structure, and pathway) information.	99.62

Supplementary figure(s)

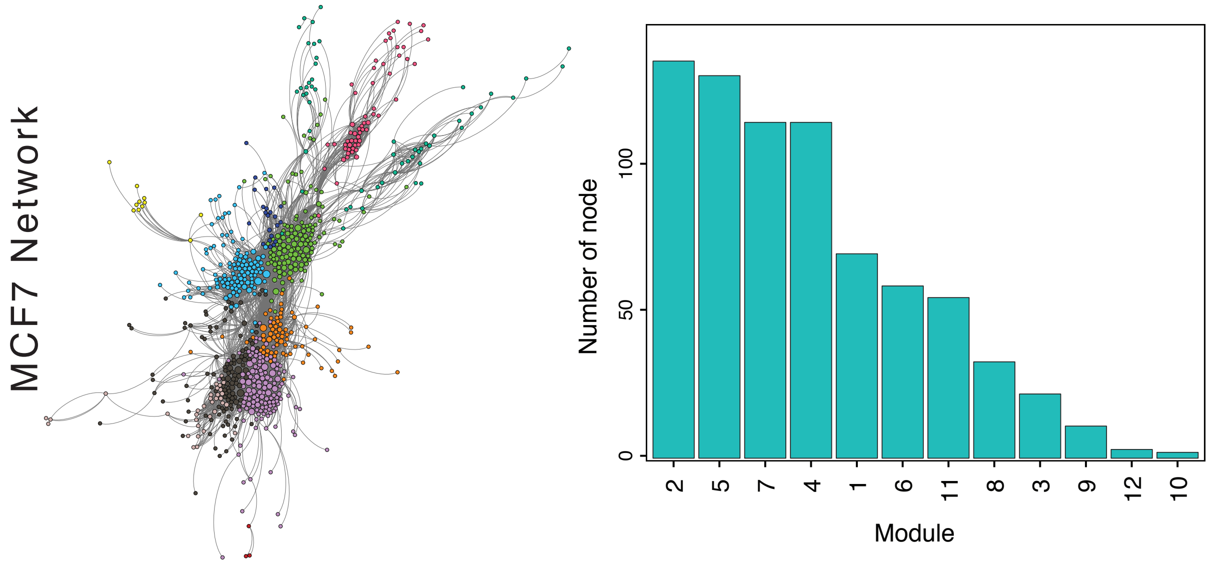

Figure 1: Drug network connecting the most associative drugs using JI and module annotation for MCF7 cell line (Left). The network representation displays drugs as circles (nodes) connected with edges. The colour of drug corresponds to their associative grouped module. The bar chart shows the number of nodes in each module of the cell line networks (Right).

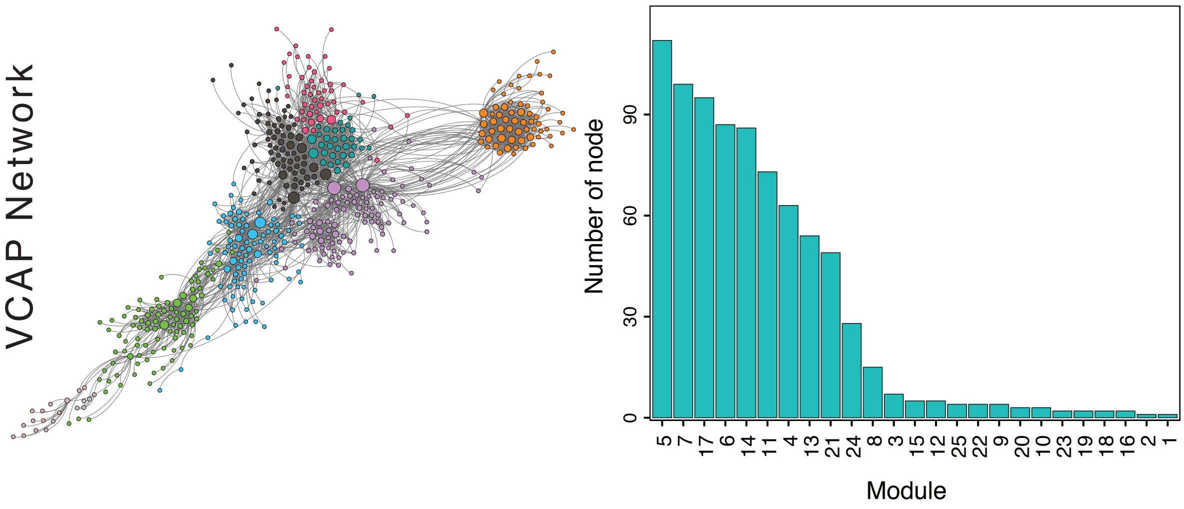

Figure 2: Drug network connecting the most associative drugs using JI and module annotation for VCAP cell line (Left). The network representation displays drugs as circles (nodes) connected with edges. The colour of drug corresponds to their associative grouped module. The bar chart shows the number of nodes in each module of the cell line networks (Right).

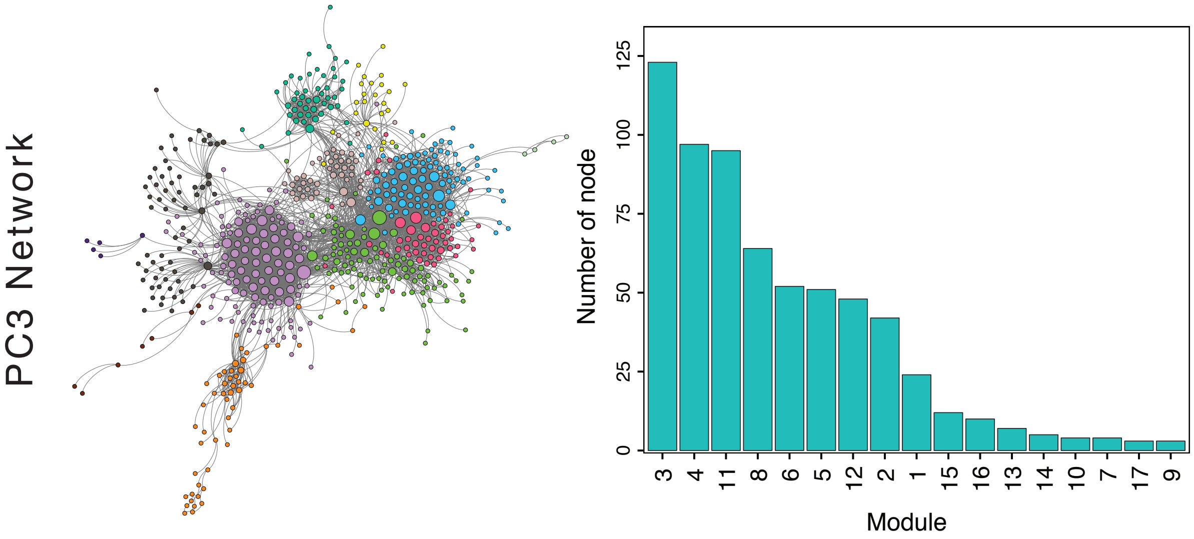

Figure 3: Drug network connecting the most associative drugs using JI and module annotation for PC3 cell line (Left). The network representation displays drugs as circles (nodes) connected with edges. The colour of drug corresponds to their associative grouped module. The bar chart shows the number of nodes in each module of the cell line networks (Right).

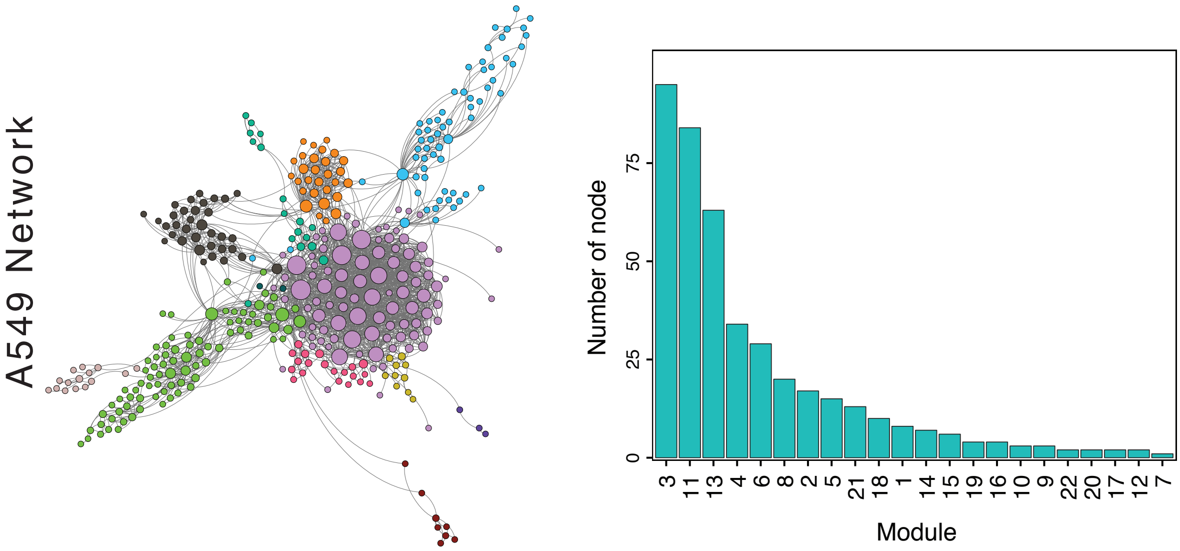

Figure 4: Drug network connecting the most associative drugs using JI and module annotation for A549 cell line (Left). The network representation displays drugs as circles (nodes) connected with edges. The colour of drug corresponds to their associative grouped module. The bar chart shows the number of nodes in each module of the cell line networks (Right).

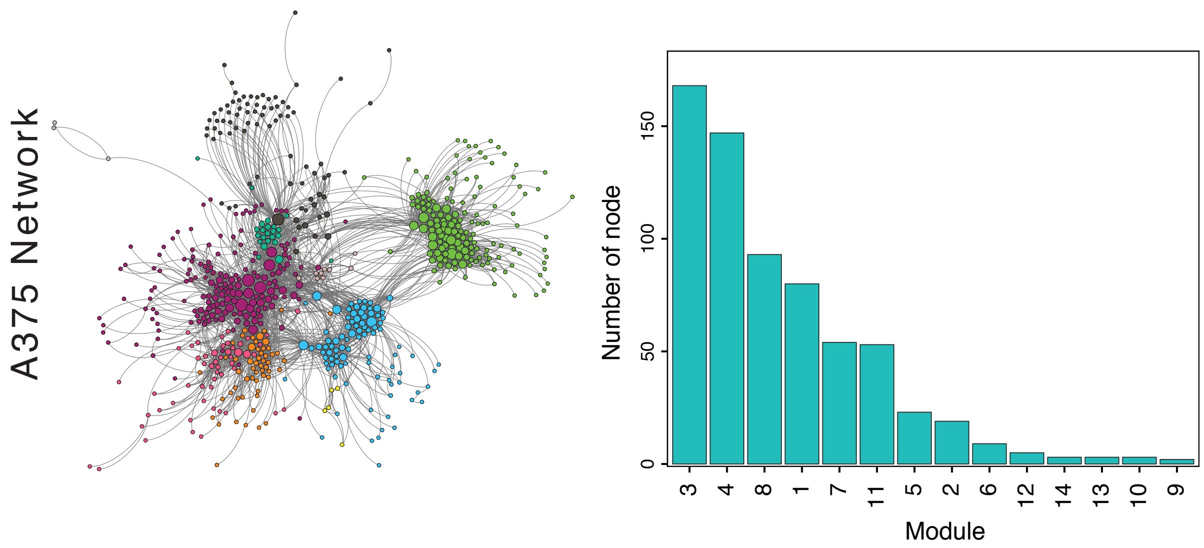

Figure 5: Drug network connecting the most associative drugs using JI and module annotation for A375 cell line (Left). The network representation displays drugs as circles (nodes) connected with edges. The colour of drug corresponds to their associative grouped module. The bar chart shows the number of nodes in each module of the cell line networks (Right).

References

[1] Evan E Bolton, Yanli Wang, Paul A Thiessen, and Stephen H Bryant. Pubchem: integrated platform of small molecules and biological activities. In *Annual reports in computational chemistry*, volume 4, pages 217–241. Elsevier, 2008.

[2] Anna Gaulton, Louisa J Bellis, A Patricia Bento, Jon Chambers, Mark Davies, Anne Hersey, Yvonne Light, Shaun McGlinchey, David Michalovich, Bissan Al-Lazikani, et al. Chembl: a large-scale bioactivity database for drug discovery. *Nucleic acids research*, 40(D1):D1100–D1107, 2011.

[3] Susumu Goto, Yasushi Okuno, Masahiro Hattori, Takaaki Nishioka, and Minoru Kanehisa. Ligand: database of chemical compounds and reactions in biological pathways. *Nucleic acids research*, 30(1):402–404, 2002.

[4] Aravind Subramanian, Rajiv Narayan, Steven M Corsello, David D Peck, Ted E Natoli, Xiaodong Lu, Joshua Gould, John F Davis, Andrew A Tubelli, Jacob K Asiedu, et al. A next generation connectivity map: L1000 platform and the first 1,000,000 profiles. *Cell*, 171(6):1437–1452, 2017.

[5] David S Wishart, Craig Knox, An Chi Guo, Savita Shrivastava, Murtaza Hassanali, Paul Stothard, Zhan Chang, and Jennifer Woolsey. Drugbank: a comprehensive resource for in silico drug discovery and exploration. *Nucleic acids research*, 34(suppl_1):D668–D672, 2006.
